# Determinants of sexual dysfunction in women with multiple sclerosis

**DOI:** 10.1186/1471-2377-13-83

**Published:** 2013-07-12

**Authors:** Khadijeh Mohammadi, Parvin Rahnama, Sakineh Moayed Mohseni, Mohammad Ali Sahraian, Ali Montazeri

**Affiliations:** 1 Department of Midwifery, Faculty of Nursing and Midwifery, Shahed University, Tehran, Iran; 2 Department Obstetrics and Gynecology, Faculty of Medicine, Shahed University, Tehran, Iran; 3 MS Research Center, Sina Hospital, Iranian Center for Neurological Research, Tehran University of Medical Sciences, Tehran, Iran; 4 Mental Health Research Group, Health Metrics Research Centre, Iranian Institute for Health Sciences Research, ACECR, Tehran, Iran

## Abstract

**Background:**

The aim of present study was to determine disease-related and psychological risk factors for sexual dysfunction in women with multiple sclerosis (MS).

**Methods:**

This was a clinical-based study conducted from September 2009 to June 2010 in Tehran, Iran. A consecutive sample of female patients with MS was recruited from an outpatient clinic. The Female Sexual Function Index (FSFI) was used to evaluate sexual function. In addition neurological impairment was measured using the Kurtzke Expanded Disability Status Scale (EDSS), and depression was assessed using the Beck Depression Inventory-II (BDI-II). Univariate and multiple logistic regression analyses were performed in order to examine the association between sexual dysfunction and independent variables.

**Results:**

In all, 226 women participated in the study. Of these, 125 women (55.3%) met the criteria for sexual dysfunction. The mean age of participants was 35.7 years (SD = 8.07). The results obtained from multiple logistic regression analysis indicated that the disease duration (OR for the disease duration of equal or greater than 9 years = 3.13, %95 CI = 1.29-7.57, P = 0.01), the disease course (OR for secondary progressive MS = 3.96, %95 CI = 1.55-10.10, P = 0.004) and the BDI score (OR = 1.11, %95 CI = 1.07-1.16, P < 0.001) were significant factors contributing to sexual dysfunction in these patients.

**Conclusions:**

The findings from this study indicated that the duration and severity of the disease in addition to depression were the most significant factors that contributed to sexual dysfunction in women with multiple sclerosis. The burden of disease and sexual dysfunction suggests the need for further attention to this patient population.

## Background

Multiple sclerosis (MS) is a chronic neurological disorder that is characterized by disseminated demyelination of nerve fibers of the brain and spinal cord [1]. MS affects both male and female gender, but the disease affects women 2 to 3 times more than males [2]. Thus it is not surprising that there are many women who are suffering from the disease. MS severely destroys women’s life in several aspects including disruption in sexual functioning [3-5]. The prevalence of sexual dysfunction in female patients with MS varies from 40 to 70% [6].

Several studies demonstrated that different risk factors contribute to the development of female sexual dysfunction in MS patients including presence of physical disorders, neurological impairments, age at onset of the disease, depression and anxiety [3,7-10].

The prevalence of MS in Iranian population is estimated to be at least 51.9 per 100,000 [11]. Also, female-to-male ratio for the disease is reported to be 3.11 [12]. Overall sexual dysfunction among Iranian females has been reported in up to 31% of women [13], but to the best knowledge of the authors prevalence of sexual dysfunction in Iranian women with multiple sclerosis is unknown.

Sexuality is a sensitive issue in almost every culture including Iran. Thus patients usually are reluctant to talk about their sexual problems and often, sexual dysfunction goes under recognized and under treated. If female sexuality is disturbed then it might lead to several problems, even divorce and family breakdown [14]. It also affects reproductive health and family planning. The aim of present study was to determinant disease-related and psychological risk factors for sexual dysfunction in women with multiple sclerosis (MS) in order to recognize the extent of the problem and perhaps provide appropriate guidelines for planning managed care for these patients.

## Methods

### Design and procedure

This was a clinical-based study conducted in Tehran, Iran, during a 9-months period from September 2009 to June 2010. A consecutive sample of 226 female patients with multiple sclerosis was recruited from the MS outpatient clinic in a large teaching and referral hospital affiliated to Tehran University of Medical Sciences. Criteria for inclusion were: diagnosis of MS according to the McDonald Revised criteria [15]; being married; having Expanded Disability Status Scale (EDSS) score less than or equal to 8 [16], and willingness to participate in the study. Exclusion criteria were: pre-existing major chronic illness, and not having sexual experience in life. All patients had a full neurologic examination.

### Questionnaires

We used several questionnaires to collect data:

1. A study specific questionnaire in order to collect data on demographic, clinical, and obstetric information.

2. The Female Sexual Function Index (FSFI) was used to evaluate sexual function [17]. The FSFI is a validated and widely used 19-items self-reported measure of women’s sexual function including the following dimensions: desire, arousal, lubrication, orgasm, satisfaction, and pain. The psychometric properties of the Iranian version of Female Sexual Function Index are well documented [18]. Fortunately the authors performed sensitivity analysis for the Iranian version and the cut-off point for the scale was found to be 28 (sensitivity = 83% and specificity = 82%). In addition the same study carried out the Receiver Operating Characteristics analysis (ROC) and reported that the Area under the Curve (AUC) was 0.917. The study used the clinical diagnostic as the gold standard and reported that 28 was the best cut-off value for predicting sexual dysfunction. Thus, for the analysis purpose women who scored equal or less than 28 on the FSFI were identified as those who were suffering from sexual dysfunction. Similarly the cut-off points for the subscales also were indicated and reported as follows: Desire = 3.3, Arousal = 3.4, Lubrication = 3.7, Pain = 3.8, Orgasm = 3.4, Satisfaction = 3.8.

3. Depression was assessed using the Beck Depression Inventory-II (BDI-II) [19]. It is a widely used 21-items self-reported measure that assesses the presence and intensity of depressive symptoms reflecting the similar symptoms suggested by the Diagnostic and Statistical Manual of Mental Disorders (DSM-IV) [20]. The Persian version of inventory already has been validated in Iran [21].

4. The neurological impairment was assessed using the Expanded Disability Status Scale (EDSS). The EDSS is a gold standard for assessment of disability in people with MS. A neurologist scored the EDSS for each patient [16]. The score on the EDSS ranges from 1 to 9.5 where scores from 1.0 to 4.5 specify that patients are fully capable of walking while higher scores indicate that patients are severely impaired.

### Analysis

Descriptive analysis was carried out to explore the data. Participants were classified as with and without sexual dysfunction based on the FSFI cut-off score as mentioned earlier. We used both univariate and multiple logistic regression analyses to examine the association between dependent (sexual dysfunction) and independent variables. The level of significance was set at 5%. The SPSS version 16 was used to analyze the data.

### Ethics

The ethics committee of Shahed University approved the study. We obtained written informed consent from participants after comprehensive explanation of procedure involved.

## Results

### Demographic and clinical characteristics of the study sample

In all, 320 patients were approached and 226 participants have agreed to participate in the study, giving a response rate of 71%. Overall, 125 women (55.3%) met the criteria for sexual dysfunction. The mean age of participants was 35.7 years (SD = 8.07). The mean disease duration of participants was 1.8 (SD = 0.79) years, and had the following diagnosis: 169 patients (74.8%) had relapsing remitting MS (RRMS), 4 women (1.8%) had primary progressive MS (PPMS) and 53 patients (23.5%) had secondary progressive MS (SPMS). Frequency of sexual dysfunction was 49% in RRMS, 75% in SPMS and 50% in PPMS. The detailed results are shown in Table 1.

**Table 1:** Characteristics of the study sample and odds ratio for sexual dysfunction (n = 226)

	**With SD (n = 125)**	**Without SD (n =101)**	OR (95% CI)*	
	**No. (%)**	**No. (%)**		**P**
**Age (years)**				
≤ 35	54 (43.2)	63 (62.4)	1.0 (ref.)	
> 35	71 (56.8)	38 (37.6)	2.18 (1.27–3.72)	0.004
**Education**				
Higher	28 (22.4)	41 (40.5)	1.0 (ref.)	
Primary/Secondary	97 (77.6)	60 (59.5)	2.36 (1.32–4.22)	0.003
**Employment status**				
Employed	13 (10.4)	24 (23.8)	1.0 (ref.)	
House wife	112 (89.6)	77 (76.2)	2.68 (1.28–5.59)	0.008
**Duration of marriage (years)**				
>10	45 (36.0)	51 (50.5)	1.0 (ref.)	
10–20	45 (36.0)	36 (35.6)	1.41 (0.78–2.56)	0.250
21–40	35 (28.0)	14 (13.9)	2.83 (1.35–5.92)	0.006
**Disease duration (years)**				
0-8	82 (65.6)	87 (86.1)	1.0 (ref.)	
≥ 9	43 (34.4)	14 (13.9)	3.25 (1.66–6.39)	0.001
**Disease course**				
RRMS	83 (66.4)	86 (85.1)	1.0 (ref.)	
PPMS	2 (1.61)	2 (2.0)	1.03 (0.14–7.52)	0.972
SPMS	40 (32.0)	13 (12.9)	3.18 (1.59–6.83)	0.001
**EDSS score**				
0–4.5	82 (65.6)	83 (82.2)	1.0 (ref.)	
5–8	43 (34.4)	18 (17.8)	2.41 (1.28–4.53)	0.006
**BDI score** (mean/SD)	22.20 (10.94)	12.48 (8.79)	1.11 (1.07–1.15)	< 0.0001

* Derived from univariate logistic regression analyses while sexual dysfunction was considered as dependent variable and other characteristics were considered as independent variable.

### Risk factors for sexual dysfunction

The association of sexual dysfunction and independent variables was first examined by univariate logistic regression analysis. The results showed that there were significant associations between sexual dysfunction and age (OR for 36–70 years = 2.18, %95 CI = 1.27-3.72), education (OR = 2.42, %95 CI = 1.34-4.37), employment status (OR for housewife = 2.68, %95 CI = 1.28-5.59), duration of marriage (OR for 21–40 years = 2.83, %95 CI = 1.35-5.92), the disease duration (OR for ≥9 years = 3.25, %95 CI = 1.66-6.39), the disease course (OR for SPMS = 3.18, %95 CI = 1.59-6.83), EDSS (OR for 5–8 = 2.41, %95 CI = 1.28-4.53), and the BDI (OR = 1.11, %95 CI = 1.07-1.15). The results are shown in Table 1.

All significant findings in univariate analysis then were entered into a multiple logistic regression model. As shown in Table 2 the results indicated that the disease duration (OR for ≥9 years 3.13, %95 CI = 1.29-7.57, P = 0.01), the disease course (OR for secondary progressive MS = 3.96, %95 CI = 1.55-10.10, P = 0.004) and the BDI score (OR = 1.11, %95 CI = 1.07-1.16, P < 0.001) were significant contributing factors to sexual dysfunction in these patients.

**Table 2:** The results obtained from multiple logistic regression analysis indicating risk factors for sexual dysfunction (n = 226)

		**Adjusted OR (95% CI)**	**P**
**Disease duration (years)**			
	0-8	1.0 (ref.)	
	≥ 9	3.13 (1.29–7.57)	0.01
**Disease course**			
	RRMS	1.0 (ref.)	
	PPMS	1.21 (0.15–9.69)	0.85
	SPMS	3.96 (1.55–10.10)	0.004
**BDI score**		1.11 (1.07–1.16)	< 0.001

Further analyses of the data for the subscales of questionnaire (desire, arousal, lubrication, orgasm, satisfaction, and pain) are shown in Tables 3 and 4.

**Table 3:** Descriptive statistics for sexual dysfunctions for subscales of the FSFI (n = 226)

		**No (%)**	**Cut-off point**
**Desire**			3.3
	Yes	77 (34.1)	
	No	149 (65.9)	
	Mean score (SD)	3.68 (1.38)	-
**Arousal**			3.4
	Yes	100 (44.2)	
	No	126 (55.8)	
	Mean score (SD)	3.38 (1.75)	-
**Lubrication**			3.7
	Yes	41 (18.1)	
	No	185 (81.9)	
	Mean score (SD)	4.88 (1.89)	-
**Pain**			3.8
	Yes	29 (12.8)	
	No	197 (87.2)	
	Mean score (SD)	4.98 (1.62)	-
**Orgasm**			3.4
	Yes	81 (35.8)	
	No	145 (64.2)	
	Mean score (SD)	3.77 (1.87)	-
**Satisfaction**			3.8
	Yes	54 (23.9)	
	No	172 (76.1)	
	Mean score (SD)	4.50 (1.29)	-

**Table 4:** The results obtained from the logistic regression analyses for sexual dysfunctions for subscales of the FSFI (n = 226)*

		**Adjusted OR (95% CI)**	**P**
*Desire*			
	**Age**		
	≤ 35	1.0 (ref.)	
	> 35	2.06 (1.14–3.69)	0.015
*Arousal*			
	**Age**		
	≤ 35	1.0 (ref.)	
	> 35	3.18 (1.80–5.62)	< 0.001
*Lubrication*			
	**Age**		
	≤ 35	1.0 (ref.)	
	> 35	2.82 (1.34–5.92)	0.006
*Pain*			
	**Age**		
	≤ 35	1.0 (ref.)	
	> 35	4.58 (1.77–11.80)	0.002
	**Duration of marriage (years)**		
	>10	1.0 (ref.)	
	10-20	2.84 (0.78–2.56)	0.042
	21-40	2.92 (1.35–5.92)	0.113
*Orgasm*			
	**Age**		
	≤ 35	1.0 (ref.)	
	> 35	2.55 (1.41–4.61)	0.002
	**Disease duration (years)**		
	0-8	1.0 (ref.)	
	≥ 9	0.42 (0.19–0.93)	0.033
	**BDI score**	1.03 (1.00–1.06)	0.023
*Satisfaction*			
	**Age**		
	≤ 35	1.0 (ref.)	
	> 35	3.44 (1.70–6.96)	0.001
	**EDSS score**		
	0-4.5	1.0 (ref.)	
	5-8	0.33 (0.11–0.95)	0.04
	**BDI score**	1.04 (1.00–1.07)	0.012

* Derived from multiple logistic regression analysis while dysfunctions on the above subscales were treated as dependent variables and age, education, occupation, disease duration, marriage duration, disease severity, the EDSS and the BDI scores were considered as independent variables. Only the significant results are reported for each subscale.

## Discussion

The findings from this study indicated that considering the cut-off point of 28 on the FSFI, the prevalence of sexual dysfunction among Iranian female with MS was high (55.3%). However, one might argue that most studies chose a cut-off point of lower than 26.55 for differentiating women with and without sexual dysfunction [22]. Then, our findings would have been decrease slightly. The findings also showed that there was no significant association between sexual dysfunction and age. In fact, although age was significant factor in univariate analysis (see Table 1), its effect was diminished when performing multiple logistic regression analysis. This observation might be explained by the fact that our sample consisted of women who perhaps were less sexually active. Another reason for such observation might be related to the small sample size of the study. Studying 73 MS patients a similar finding was reported by Khan et al. where they have found no association between age and sexual dysfunction as measured by the Sexual Frequency Scale [23].

The results of this study showed that duration of the disease was associated with sexual dysfunction. This implies that the more time passes, increased sexual dysfunction will occur. A study showed that the extent and the number of sexual dysfunction symptoms increased significantly in MS patients during 2-years follow-up [24]. Similarly another study revealed that duration of the disease in MS patients who suffered from sexual dysfunction was longer than the duration in MS patients without SD [25]. This might be explained by several reasons including the fact that MS could have a negative impact on relationship between patients and their sexual partners [26], and that the high levels of stress among patients’ partners might affect their emotional and sexual functioning [27].

The results of this study confirmed the previous reports where it has been shown that SD was associated with progressive forms of MS [26,28]. It seems that there is a very straightforward relationship between sexual dysfunction in women with MS and progressive course of the disease.

This study also showed that depression was associated with sexual dysfunction in this population. Depression in MS is a multidimensional problem that varies by disease related impairments, activity restrictions and unpredictable prognosis [29,30]. Also, in patients with MS it is significantly higher than healthy individuals [31,32] and is considered as a common co-morbidity among patients with multiple sclerosis [33,34]. Consistent with our findings, other studies have reported that there was significant correlation between presence of sexual dysfunction and depression [9,28,35]. It has been suggested that depression may be a prominent variable contributing to the sexual difficulties in MS patients. In fact depressed women might suffer from several symptoms including affective and somatic symptoms. These symptoms all contribute to female sexual dysfunction since they might cause mood and desire disorders [36,37]. In addition, women with depression might use medication and the relationship between antidepressant medications and sexual dysfunction are well documented [38]. Studies have shown that sexual dysfunction commonly occurs during antidepressant treatment and considered as common adverse effect of antidepressant treatment [39].

### Limitations

This study had some limitations. It was a cross-sectional study. Also we obtained our sample from an outpatient clinic. Thus, the results from this study must be interpreted with caution.

## Conclusion

The findings indicated that sexual dysfunction was frequent in women with multiple sclerosis. The findings also indicated that clinical and psychological factors were the most important contributing variables to sexual dysfunction in women with multiple sclerosis. The burden of sexual dysfunction in addition to the disease burden suggests the need for extra attention to this patient population.

## Competing interests

The authors declare that they have no competing interests.

## Authors’ contributions

KM was the main investigator and involved in the study design, data collection and writing process. SMM contributed to the writing process. MAS contributed to the study design and recruitment of patients. PR analyzed the data and wrote the paper. AM critically evaluated the paper, helped in analysis, responded to the reviewers’ comments and provided the final manuscript. All authors read and approved the final manuscript.

## Pre-publication history

The pre-publication history for this paper can be accessed here:

http://www.biomedcentral.com/1471-2377/13/83/prepub
